# Supplementary material for: Are carbon emissions trading and green financial instruments synergistic? -Comprehensive quantitative research based on content analysis
Source: PLoS One. 2024 Mar 7;19(3):e0298601. doi: 10.1371/journal.pone.0298601 (PMC10919601; doi:10.1371/journal.pone.0298601)
Supplement: S1 Data — (ZIP) [file pone.0298601.s001.zip › Data/地级市Z_F工作报告.zip!/╔╜╢1⁄2/╟α╡║╩╨/└┤╫╘╣1⁄2╓┌║┼í╛┬φ┐╦╩2╛▌═°í┐_2015.docx]

各位代表：

　　现在，我代表市人民政府向大会报告工作，请予审议，并请各位政协委员和其他列席人员提出意见。

　　一、2014年工作回顾

　　过去的一年，面对复杂严峻的外部环境和艰巨繁重的发展任务，在中共青岛市委的领导下，我们团结依靠全市人民，全面贯彻党的十八大和十八届三中、四中全会精神，深入贯彻习近平总书记系列重要讲话精神，积极作为，真抓实干，统筹推进稳增长、促改革、调结构、惠民生，全市呈现经济平稳发展、民生持续改善、社会和谐稳定的良好局面。

　　全市生产总值实现8692.1亿元，增长8%；一般公共预算收入实现895.2亿元，增长13.5%；固定资产投资增长16.1%；社会消费品零售总额增长12.6%；城乡居民收入分别增长8.7%和11%；城镇登记失业率2.97%；居民消费价格上涨2.6%；人口自然增长率5‰；完成了节能减排各项任务。

　　（一）着力扩内需稳外需，经济实现稳定增长

　　积极应对经济下行压力，促进投资消费出口协调拉动增长。深入实施调结构、稳增长、抓落实现场推进行动，开展走访千家企业、走进千个项目现场、走谈千个项目活动。200个市级重点建设项目全部开工，竣工70个，完成投资1560亿元。一汽大众华东生产基地等一批重大项目顺利推进。实施十大消费领域拓展工程，积极培育信息消费、休闲旅游等新兴消费。接待国内外游客和旅游总收入分别增长8.8%、15%。实施国际贸易中心城市建设“五大行动计划”，实现进出口总额799亿美元,其中出口增长9.1%。服务贸易增长28.7%。

　　坚持综合施策，优化企业发展环境。出台促进民营经济、小微企业、新型业态发展的系列政策，完善大企业直通车制度和中小企业公共服务体系。对省级以上开发区实行项目落地审批零收费。落实结构性减税政策，依法减免、暂停、后置征收69项行政事业性收费，为企业减负61.3亿元。多措并举缓解小微企业融资难问题，小微企业贷款增长10.6%，连续三年高于各项贷款增速。

　　（二）着力抓改革促开放，发展活力不断增强

　　重点领域改革实现突破。市、区（市）两级政府机构改革基本完成，市政府工作部门由46个减至38个，机关事业单位编制进一步精简。实施工商登记制度改革，前置审批由381项减为37项，市场主体总量和注册资本总额分别增长53.9%、27.9%。县域行政综合执法改革试点取得明显成效。创新财政专项资金运作方式，放宽社会资本投资领域，民间投资增长24%。财富管理金融综合改革试验区建设顺利推进。培育引进金融机构12家，中路财险公司获批筹建。蓝海股权交易中心挂牌企业131家。青岛港等3家企业上市。农村土地“三权”分置改革顺利推进。农村“四权”担保融资累计发放贷款突破1亿元。出台户籍制度改革意见，实施积分落户政策。

　　开放水平进一步提升。积极复制推广上海自贸区经验，投资贸易便利化改革试点有序推进。多式联运海关监管中心建成运营。引进惠普全球大数据中心等一批外资大项目，实际利用外资60.8亿美元，增长10.2%。引进内资1380亿元，增长13.5%。对口支援工作扎实推进。纳入国家“一带一路”战略规划，被确定为新亚欧大陆桥经济走廊主要节点城市和海上合作战略支点城市。对外投资额增长24.6%。新加坡青岛工商中心挂牌运营。中韩贸易合作区建设工作全面启动。成为中美地方经贸合作重点城市。与法国孚日省结为友好合作关系城市。与德国汉堡等10个国外城市建立经济合作伙伴关系。泰国驻青总领馆开馆。成功承办亚太经合组织贸易部长会议和第二次高官会。西太平洋海军论坛顺利举行。

　　（三）着力调结构促转型，转变发展方式取得新进展

　　产业结构进一步优化。海洋生产总值增长13.1%，占全市生产总值比重达到20.2%。工业十条千亿级产业链产值增长10.2%。高端服务业十个千万平米工程竣工2516万平方米。服务业增加值占全市生产总值比重达到51.2%，提高1.1个百分点。推出十大新兴产业发展规划，战略性新兴产业增加值增长20.5%。粮食总产量达到323万吨。远洋捕捞产量翻两番。成功举办第十二届中国国际农产品交易会。

　　产业布局加快调整。西海岸新区获批成为第九个国家级新区，为青岛转型跨越发展带来重大机遇。黄岛石化区功能规划调整启动实施。蓝色硅谷成为国家科技兴海产业示范基地。红岛经济区获批国家海洋生物医药特色产业基地。市南总部经济中心区、市北中央商务区、李沧现代商贸区、崂山金融聚集区、城阳轨道交通产业区加快建设。实施腾笼换鸟、凤凰涅槃行动方案，老城区工业企业累计启动搬迁104户，竣工投产50户。即墨汽车产业新城、胶州中铁集装箱中心、平度南村家电城、莱西新能源汽车产业基地等县域工业园集聚效应凸显。四市规模以上工业总产值占全市比重达到46.7%，提高2.1个百分点。

　　自主创新能力不断增强。加强与中科院、清华大学、哈尔滨工程大学、中船重工等院企合作，引进建设一批产学研机构、产业化基地。海洋科学与技术国家实验室投入试运行。新增市级以上企业技术中心97家、工程技术研究中心30家。科技孵化器累计投入使用443万平方米，入驻企业达到2816家。新引进人才11.7万人。获批建设国家首个海洋技术转移中心。发明专利申请量和授权量分别增长21.5%、48.4%。技术合同交易额增长70.9%。启动实施“千帆计划”，扶持科技型中小企业发展。高新技术产业产值占规模以上工业产值比重达到40.7%。

　　（四）着力抓统筹促均衡，城乡一体化发展取得新成效

　　重大基础设施加快建设。胶东国际机场获得国务院和中央军委批复，建设工作全面启动。董家口港区加快建设，全市港口吞吐量和集装箱吞吐量分别达到4.8亿吨和1658万标箱。地铁2号、3号线进展顺利，青荣城际铁路即墨至荣成段开通。智慧青岛加快建设，成为中国领军智慧城市。

　　新型城镇化有序推进。新一轮城市总体规划修编完成并上报国务院。城市地下空间资源综合利用规划编制完成。成为国家新型城镇化综合试点城市。新型农村社区累计开工304个，建成117个，24.5万农民搬进新居。实施小城镇环境综合整治，初步建立城乡一体化垃圾收集处理机制。实施23处农村规模化供水工程，人口覆盖率达到85%。完成1326个村电网治理。县乡公路、中小河道治理、农田水利工程建设取得新进展。

　　生态建设成效明显。大沽河治理工程全面竣工，展现出洪畅、堤固、水清、岸绿、景美的母亲河新形象。推进环胶州湾流域截污治污工程，加强城市河道和农业面源污染治理，污水处理厂、排污管网和污水直排口整治等工程顺利实施。建成万亩林场10个，国家森林城市创建通过验收。成功举办一届精彩、惠民、有特色、高水平的世界园艺博览会。

　　（五）着力惠民生促和谐，民生质量持续改善

　　市级财政安排民生支出198.3亿元，占财政支出比重达到65.7%，提高1.7个百分点。新增5项民生保障政策，拓展5项民生保障范围，提高10项民生保障标准。为城乡居民重点办好的10件实事全部完成。

　　民生保障水平进一步提高。新建、改扩建幼儿园177所，新建成16所中小学。311所中小学标准化食堂建成使用。义务教育学校校长教师轮岗交流制度全面推行。经济欠发达镇教育扶助计划顺利实施。安排了13万名新市民子女市区就学。重点建设项目与就业联动机制、劳动力资源管理信息系统初步建立，培训劳动力10万人次，新增就业44.5万人，扶持创业2.4万人。市第三人民医院建成使用，社会办医院新增19家，新增医疗床位2000张。“单独两孩”政策平稳实施。区市8处社会福利中心投入运营。政府为困难老人购买居家养老服务实现全覆盖。加大困难群众救助力度，为15.6万低保户发放保障金7.8亿元。城乡困难居民医疗救助标准实现均等化。为1.9万名重度残疾人发放护理补贴，为3万名残疾人实施康复服务。启动主城区棚户区改造7665户，筹建保障性住房17344套，配租配售9443套。改造农村危房3000户。市区新增集中供热面积550万平方米，其中清洁能源供热200万平方米。完成2.1万户城市居民用电“一户一表”改造。

　　实施六大领域专项治理。严格落实安全生产目标责任，持续加强对重点行业领域的专项整治和隐患排查。强化农业投入品监管，健全了农药监管、追溯、检测和执法体系。查处食品药品安全案件4987件，食品抽检合格率进一步提高。强化空气污染治理，市区空气细颗粒物（PM2.5 ）年均浓度下降10.6%。加强铁路沿线等重点区域环境整治，市容环境得到改善。建成智能交通系统一期工程，新增公共停车泊位2.9万个。加大违法犯罪活动打击力度，组建了反恐处置突发事件特警机动队伍，打造立体化治安防控体系，视频监控“天网”工程覆盖率达到80%，社会环境保持安定有序。全面加强应急管理，提升了全社会应急能力。畅通信访渠道，将涉法涉诉信访问题纳入法治轨道解决。

　　城市文明水平进一步提升。扎实开展第四轮全国文明城市创建工作。公共文化设施开工在建20.3万平方米。4部文艺作品荣获全国“五个一工程”奖和鲁迅文学奖。“扫黄打非”和文化市场管理成效明显。广泛开展全民健身运动，青岛籍运动员在第17届亚运会、第23届省运会上取得优异成绩。双拥共建持续加强，军政军民关系更加融洽。统计、档案、史志工作取得新成效。法律援助和服务水平不断提升，仲裁作用进一步发挥。侨务、对台和民族宗教工作得到加强。人防、气象、防震减灾工作有序开展。妇女儿童、青少年、老龄工作成效明显。红十字、残疾人、慈善事业健康发展。

　　（六）着力转作风提效能，政府自身建设不断加强

　　依法行政水平进一步提高。自觉把政府工作置于人大及其常委会监督之下，认真落实人大及其常委会决议决定，主动向人大报告重大事项，积极提请并全力配合专题视察，自觉接受专题询问。在重大问题决策前、决策中加强民主协商，积极支持政协履行政治协商、民主监督和参政议政职能。市人代会大会议案办理取得阶段性成果，642件市人大代表建议、698件市政协提案全部按期办复。多渠道加强与人大代表、政协委员的联系服务工作，广泛听取各民主党派、工商联、无党派人士的意见建议。向市人大常委会提请审议地方性法规草案5件，制定政府规章5件。

　　勤政廉政建设扎实推进。深入开展党的群众路线教育实践活动，毫不放松抓好“四风”问题整改和庸懒散慢拖瞒治理，实施作风效能集中整治专项行动，健全服务基层、政民互动的长效机制，政风建设取得积极成效。落实政府廉政建设责任，加强行政审批、行政执法、工程建设、公共资源交易等重点领域廉政建设。深化政务公开，市级行政审批事项目录发布。建立了统一规范的公共资源交易平台和管理体制。市本级“三公”经费预算减少10.4%。加强行政监察和审计监督，严肃查处一批违法违纪案件，推进了反腐倡廉建设，努力营造风清气正的政务环境。

　　过去一年所取得的成绩，是党中央国务院、省委省政府和市委科学决策、正确领导的结果，是市人大、市政协和社会各界有效监督、大力支持的结果，是全市人民团结拼搏、不懈奋斗的结果。在此，我代表青岛市人民政府，向全体市民和来青建设者，向人大代表和政协委员，向中央、省驻青单位，向驻青部队和武警官兵，向社会各界人士和离退休老同志，向所有关心支持青岛发展的海内外朋友们，表示衷心的感谢和崇高的敬意!

　　各位代表，从去年的工作看，青岛的发展还面临许多困难和问题。经济增长下行压力较大，一些重点项目进展不快，过亿元大项目开工数量减少，批发零售业销售额、住宿餐饮业营业额增速回落，中小企业经营成本上升、订单减少、融资困难。新兴产业对经济增长的支撑力不够强，科技成果转化率不够高，淘汰落后和化解过剩产能的任务较重。房地产市场变化影响较大，结构性矛盾比较突出。非法集资、逃废银行债务问题显现，银行业金融机构不良贷款率上升。社会诚信体系还不健全，市场秩序有待规范。项目用工需求和劳动力培训就业衔接得还不够密切。社会保障水平有待提高，一些因病致贫群众的生活状况艰难。棚户区改造一些片区进展不快。农村镇和社区教育资源配置不足，学校办学条件还不完善。医疗卫生事业发展不均衡，农村社区医疗服务比较薄弱。食品药品安全工作还存在许多薄弱环节。交通拥堵、停车难问题仍然突出。大气环境改善任务很重，水污染治理还不到位。安全生产隐患多、事故多发的状况没有根本改变。影响群众安全感的侵财类案件仍然多发。一些群众来信来访反映的问题处理不及时，解决不彻底。政府职能转变还不到位，服务能力需要加强。有的干部存在不思进取、为官不为等现象，部分工作人员履职能力和依法行政水平有待进一步提高，“四风”问题和一些领域的腐败现象仍然存在。对这些问题，我们将高度重视，采取有力措施，认真加以解决。

　　二、2015年主要工作安排

　　2015年是全面深化改革的关键之年，是全面推进依法治国的开局之年，也是全面完成“十二五”规划的收官之年。当前，我国经济社会发展进入新常态，青岛正处于转型发展的关键时期。做好今年工作，我们必须强化战略思维、底线思维和机遇意识，把握好稳与进、转与接、新与旧、舍与得、出与进、面与点的关系，必须毫不动摇坚持以经济建设为中心，切实抓好发展这个第一要务，勇于担当，攻坚克难，在新常态下实现更大作为，努力在全面建成小康社会进程中走在前列，为建设经济文化强省作出更大贡献。

　　今年政府工作的总体要求是：全面贯彻党的十八大和十八届三中、四中全会精神，坚持以邓小平理论、“三个代表”重要思想、科学发展观为指导，深入学习贯彻习近平总书记系列重要讲话和视察山东重要讲话、对山东工作重要批示精神，坚持稳中求进工作总基调，以提高经济发展质量和效益为中心，主动适应经济发展新常态，把转方式调结构放到更加重要位置，深化改革开放，突出创新驱动，加强民生保障，强化依法治理，保持经济平稳健康发展和社会和谐稳定，在建设宜居幸福的现代化国际城市进程中谱写新的篇章。

　　主要预期目标是：全市生产总值增长7.5%左右，一般公共预算收入增长9.5%，固定资产投资增长15%，社会消费品零售总额增长12%，外贸出口增长5%，城乡居民收入分别增长8.5%和10%，城镇登记失业率控制在4%以内，居民消费价格涨幅控制在3%以内，人口自然增长率控制在7.2‰以内，完成“十二五”节能减排任务。

　　（一）保持经济稳定增长，提高发展质量和效益。加快“转、调、创”步伐，加大腾笼换鸟、凤凰涅槃力度，积极培育新的增长点和动力源，推动经济提质增效升级。

　　增强内需活力，促进投资消费均衡拉动增长。积极扩大有效投资，抓好总投资6400亿元的200个市级重点项目建设，竣工65个以上，完成投资1300亿元以上。加强政策引导，消除投资障碍，扩大民间投资。大力培育新的消费热点，扩大养老健康家政、信息、旅游、住房、绿色、教育文化体育等领域的消费。鼓励发展商业、科技、旅游、文化创意类地产，促进房地产业转型发展。创建国家旅游综合改革试点城市,大力发展入境游、海上游和冬季游，推动旅游业向质量效益型转变。鼓励发展电子商务，推动线上线下融合发展。壮大中介服务业规模。发展会展经济。加强现代商贸流通体系建设，做好城市共同配送等试点工作。探索农产品批发市场公益化改革，加快“菜篮子”工程升级改造。开展进口商品展、旅游惠民月等消费促进活动，推动批发零售、住宿餐饮业发展，打造城市购物节国际时尚消费品牌。

　　坚持蓝色高端新兴导向，推动产业向中高端迈进。加快海洋特色产业园建设，推进100家重点企业发展，壮大海工装备、海洋生物医药、海水综合利用等产业规模。促进信息化与工业化深度融合，调整优化工业十条千亿级产业链，推进一汽大众华东生产基地等300个转型升级项目建设，力争100个项目竣工投产。加快青钢、海晶、碱业等企业搬迁步伐，鼓励城区布局分散的工业企业转型发展。继续做好化解过剩产能、淘汰落后产能工作。抓好高端服务业十个千万平米工程，推进国际创新园二期等100个重点项目建设，争创国家级服务业集聚示范区和物流园区。落实十大新兴产业发展规划，推进中国移动青岛云计算基地等150个重点项目建设。促进信息技术与传统产业融合，鼓励互联网工业创新发展，加快大数据开发应用、智能化制造、移动互联网、云计算等新兴产业发展。

　　加快创新型城市建设,提升创新驱动力。以海纳百川、求贤若渴的气度，更加重视人才引进、培养和使用，完善人才政策，营造良好环境，增强对各类人才的吸引力。加快千万平米孵化器、千万平米软件园、千万平米人才公寓建设，构建一体化人才公共服务体系。加快公共技术平台、企业技术中心和工程技术研究中心建设，构建以企业为主导的产业技术创新战略联盟。推动惠普全球大数据中心、中船重工海工装备研究院等项目建设。深入实施“千帆计划”，壮大科技型中小企业群体。鼓励发展技术转移、科技咨询、科技金融等科技服务业，建设国家海洋技术转移中心，组建海洋科技成果转化基金。实施名牌、标准化和质量强市战略，强化知识产权保护与运用。

　　加快一谷两区创新能力建设，增强辐射带动效应。西海岸新区，实施新区建设发展三年行动方案，加快经济技术开发区转型升级，推动中德生态园向标准化、智能化、生态化园区迈进，争取设立古镇口军民融合创新示范区。蓝色硅谷核心区，推动海洋科学与技术国家实验室科研平台建设，加快国家深海基地、山东大学青岛校区等项目建设，提高科技研发、成果转化和产业培育能力。红岛经济区，加快建设研发资源集聚区，壮大软件信息、高端智能制造、蓝色生物医药、海工装备研发、新材料等主导产业规模，打造科技人文生态新城。

　　（二）全面深化改革开放，不断释放发展活力。以政府自身革命带动重要领域改革，打好全面深化改革攻坚战，再创开放型经济新优势，在新一轮改革开放中争当领头雁、火车头、排头兵。

　　推进政府职能转变。进一步削减下放市级行政审批事项，依法全面清理非行政许可审批事项。推进行政审批全流程改革，提高审批效能。深化商事制度改革，营造鼓励大众创业、万众创新的良好环境。加强监管能力建设，提高事中事后监管水平。深化行政执法体制改革，在试点基础上推广综合执法，推进执法重心下移。扩大公共资源交易范围，完善土地、政府工程、政府采购等公共资源网上交易办法和流程。整合建立统一的不动产登记管理体制。有序推进事业单位分类改革，实施学校、科研院所、医院等单位去行政化改革。

　　深化财税体制改革。出台财税体制改革意见，深化预算管理制度改革。逐步取消财政专项资金对竞争性领域的直接投入，更多采取市场化方式运作。建立专项资金管理清单制度，推进专项资金公开。发展财源经济，增强聚财能力。规范清理行政事业性收费和政府性基金。深化投融资体制改革，大力发展风险投资基金、种子基金，鼓励社会资本参与公共基础设施建设和经营。出台居民用水、用气阶梯价格政策。

　　加快金融改革发展。全面推进财富管理金融综合改革试验区建设，建立完善财富管理市场体系，促进私人银行、保险资产管理公司等新型财富管理机构集聚发展。支持地方法人金融机构发展财富管理业务。鼓励大企业发起设立或参与组建各类金融机构。做大做强青岛蓝海股权交易中心。引导企业通过上市、发行债券、私募股权等渠道直接融资。争取人民币跨境投融资等试点政策。加快引进财富管理教育科研资源，推动财富管理中介机构发展。办好2015金家岭财富论坛暨财富管理博览会。严厉打击逃废银行债务和非法集资行为，积极防范和化解金融风险，维护金融秩序稳定。

　　增强企业发展活力。推进国有企业混合所有制改革试点。加快国有小比例股权清理退出步伐。完善国有企业法人治理机制。改革国有企业经营者薪酬制度。加快国有企业上市步伐。加强国有资产监管，提高国有资本配置和运行效率。全面落实加快民营经济发展的意见，创造更加公平的市场环境。完善政银企合作机制，健全融资担保、贷款风险补偿、债券融资等扶持政策，缓解民营企业融资难问题，帮助有订单、有市场、有潜力的中小企业渡过难关。开展扶助中小微企业专项活动，落实好税费减免政策，构建万户培育、千户成长、百户升级的企业发展梯队。支持民营企业参与政府采购。更加注重发挥企业家才能，营造尊重企业家的社会氛围。

　　实施新一轮高水平对外开放。大力吸引世界500强和行业百强企业在青设立地区总部、研发中心、采购中心、结算中心，提高利用外资质量。突出发展服务贸易、转口贸易，推进跨境贸易电子商务服务试点工作，加快建设国际贸易中心城市。引导外贸企业培育自主品牌，增强国际竞争力。扩大先进技术装备、关键零部件和能源资源进口，发挥进口对结构调整的支持作用。

　　创新开放体制机制。改革外商投资管理体制，促进投资贸易便利化，打造市场化、国际化的营商环境。加快区域性电子口岸平台建设，提升多式联运海关监管水平。推动青岛自由贸易港区申建工作，支持出口加工区向综合保税区转型。加快中日韩创新产业园建设。率先融入国家“一带一路”战略，积极参与沿线国家和地区基础设施互联互通建设，开展全方位交流与合作。推进东亚海洋合作平台建设。鼓励优势企业走出去，扩大境外投资，建设境外经贸园区。坚持对外交往与经贸合作相结合,提升与国际友城的合作水平。筹建釜山等境外青岛工商中心。加强国内经济合作，做好对口帮扶工作。

　　（三）积极推进新型城镇化，促进全域统筹发展。坚持遵循规律、因势利导、顺势而为，推进中心城区、四市驻地和小城镇建设，增强城镇综合承载力，促进城镇化健康有序发展。

　　坚持规划引领。逐步推进经济社会发展、城乡建设、土地利用、生态环保等规划“多规合一”。加强控制性详规编制，提高城市设计水平。开展海洋功能区划修编工作，编制海岛保护规划。提高城市地下空间开发利用水平。完善规划调整决策机制，严格按法定程序修改规划。认真做好“十三五”规划和各专项规划编制工作。

　　促进区域协调发展。黄岛要统筹各功能区建设，加快灵山湾影视文化产业区、董家口蓝色新港城和海洋高新区建设，争创国家级旅游度假区，推进石化区功能调整。市南要加快青岛商务和时尚中心区建设，市北要展开邮轮母港城建设，李沧交通商务区要加快项目集聚，崂山金家岭金融聚集区要强化财富管理能力建设，城阳轨道交通产业区要扩大发展规模。即墨要加快建设汽车产业新城。胶州要开工建设临空经济区。平度要加快新河生态化工基地、南村家电城建设和老城区改造，建设现代化中等城市。莱西要加快建设姜山新城，打造体育休闲旅游胜地。

　　推进小城镇和新型社区建设。抓好13个小城市培育试点和省级示范镇建设，打造经济繁荣、配套齐全、环境优美、文化传承的现代化小城镇。以改善村庄面貌和农民居住条件为目标，开工建设100处各具特色的新型社区，统筹推进基础设施建设，完善社区公共服务功能。实施乡村记忆工程和传统村挂牌保护制度，打造历史文化型、传统建筑型、特色景观型、乡村旅游型村庄。改造农村危房3500户。

　　强化基础设施支撑。开工建设胶东国际机场，统筹建设综合集疏运交通体系，打造半岛区域客运枢纽。加快董家口港区码头建设。加快地铁1号、2号线和蓝色硅谷、西海岸城际轨道交通快线建设，地铁3号线北段开通运营。建成青荣城际铁路城区段，推进济青高铁和青连铁路建设，做好平度至胶州铁路连接线、至胶东国际机场轻轨项目前期工作。实施株洲路、金水路、安顺路打通工程，新疆路快速路工程主线通车，青龙高速公路青岛段建成使用。实施国省道大中修和危桥改造工程。构建连接四市、覆盖重点组团的天然气高压管网系统。推进重点输变电工程建设。完成10万户城市居民用电“一户一表”接收与改造。市区新增集中供热面积500万平方米。抓好新河水库、沐官岛水库和服务西海岸新区的调水工程建设。加快智慧青岛建设，完善信息化基础设施，光纤到户覆盖能力达到250万户。

　　（四）推动农村改革发展，提高“三农”工作水平。加大强农惠农政策力度，增强农村发展活力，加快推进农业现代化、农村公共服务均等化，促进农业基础稳固、农村和谐稳定、农民安居乐业。

　　加快转变农业发展方式。深入推进现代农业十大重点工程，促进农村一二三产业深度融合发展。落实农业补贴政策，抓好粮油高产创建，确保粮食安全。抓好大沽河两岸现代农业园区建设，鼓励发展品牌农业、休闲观光农业，整建制创建国家现代农业示范区。大力发展远洋渔业，推进国际水产品交易中心和冷链物流基地建设，打造蓝色粮仓。完善基层农技推广、疫病防控等服务体系。健全农产品流通体系，促进产销对接。加强中小河道治理、农田水利和水源工程建设，增强农业抗风险能力。加强基本农田管护，严守耕地保护红线。加大对粮食主产区、生态控制区镇村的财政转移支付力度，让为粮食生产和生态保护作出贡献的群众不吃亏、有奔头。

　　深化农村各项改革。深入推进农村土地“三权”分置改革，引导土地经营权有序流转。开展土地承包经营权“确权确股不确地”改革试点。扎实推进集体产权制度改革。完善农村“四权”抵押担保办法，健全农村产权交易市场体系。积极发展农民合作社、家庭农场、专业大户等新型农业经营主体，促进适度规模经营。推动供销社综合改革。深化农村金融体制改革，积极发展农业保险，推动金融资源向“三农”倾斜。完善户籍制度改革配套政策，健全居住证制度，有序推进农业转移人口市民化。

　　提升农村公共服务能力。继续实施经济欠发达镇教育扶助计划，加大农村师资配备和培训力度，让农村孩子享受公平有质量的教育，不输在起跑线上。完善农村基层医疗卫生服务体系，实行镇村卫生机构一体化管理，提高乡村医生待遇。加强农村社区日间照料中心建设。进一步提高农村五保供养标准。建设27处农村规模化供水工程，人口覆盖率提高到95%。完成2022个村电网升级改造。对200个贫困村、6.5万贫困人口实施精准扶贫。抓好库区移民后期帮扶工作。加强农村环境综合整治和土壤污染治理，改造农村卫生厕所7800座，新建、改扩建垃圾转运处理设施10处，新增环卫车辆200台，推动城乡环卫一体化镇村全覆盖，建设美丽乡村。

　　（五）推动文化繁荣发展，加快建设文化青岛。坚持社会主义先进文化前进方向，遵循文化发展规律，推动经济文化融合发展，增强文化软实力。

　　提高城市文明程度。深入开展社会主义核心价值观和中国梦宣传教育，大力弘扬中华优秀传统文化，挖掘利用齐鲁文化资源，增强价值观自信和文化自信。开展优良家风传承、优良校训倡导、企业精神培育、乡贤文化创新发展和学雷锋志愿服务行动，提高道德实践能力，凝聚向上向善的道德力量。深入开展群众性精神文明创建活动，启动新一轮文明城市创建工作。

　　发展文化体育事业。实施文化惠民工程，完善政府购买公共文化服务长效机制，推进国家公共文化服务体系示范区建设。扶持文艺精品创作，推出更多有筋骨、有道德、有温度的文艺作品。办好首届市民文化节、首届中国公共艺术展。加快市文化艺术中心、市博物馆等项目建设，成立青岛文化研究院和中国水彩画博物馆。做好文化遗产保护与利用工作，鼓励兴办民办博物馆、乡村陈列馆。加强文化市场管理。倡导全民健身，建设城乡居民健身设施1100处。支持足球事业发展。推进国家篮球足球学院建设。办好2015年世界休闲体育大会。

　　提升文化产业竞争力。深化文化体制改革，完善文化生产经营机制，建立现代文化市场体系。支持文化龙头企业上市。推动千万平米文化创意产业园区建设。建立影视产业发展基金，推进电影交易中心建设。积极申办青岛国际电影节，办好全国青少年小提琴比赛、世界杯帆船赛青岛站、21世纪海上丝绸之路帆船行等活动，提升影视之城、音乐之岛、帆船之都城市品牌的影响力。加快国际版权交易中心建设。发展文化贸易出口，争创国家对外文化贸易基地。办好“东亚文化之都·青岛”系列活动。

　　（六）加强生态文明建设，促进可持续发展。要像保护眼睛一样保护环境，下决心把环境污染治理好、把生态环境建设好，保持好红瓦绿树、碧海蓝天的城市特色。

　　打好治霾治水攻坚战。加大建设扬尘治理力度，淘汰全部黄标车，实施燃煤锅炉废气污染治理项目40个。全面推广新建建筑非煤化供热。实施建筑节能改造350万平方米。力争市区空气细颗粒物（PM2.5 ）年均浓度下降5%左右。加强胶州湾生态环境保护，推进陆海环境综合整治，建设环湾绿道。新建、扩建李村河、张村河、楼山河、墨水河流域污水处理厂，加快整治过城河道。巩固国家森林城市创建成果，推进生态绿化十大工程，加快浮山综合治理和生态公园建设，完善绿地生态系统，提高绿化覆盖率和容积率。

　　加强节能减排和资源集约节约利用。做好国家低碳城市试点工作。深化重点用能单位节能低碳行动。推进建筑产业化发展，提高建筑废弃物资源化综合利用水平。大力发展循环经济，推广清洁生产和超洁净排放先进技术。完善充电基础设施，加快新能源汽车推广应用。以工程减排为重点，深挖减排潜力，控制主要污染物排放总量。

　　完善生态保护机制。深入贯彻新修订的《环境保护法》，严守生态红线，严保生态环境，坚决保卫绿色财富。实施空气质量生态补偿。在大沽河流域开展水环境质量生态补偿试点。加强海岸线保护，严格海岸带设施管理，科学规划、合理开发岸线资源。争取建设碳排放权交易市场，开展排污权交易试点。实行严格的环保准入制度，加大环保执法力度，严肃查处环境违法行为。

　　（七）发展社会事业，持续保障和改善民生。坚持保基本、兜底线、促公平，持续加大民生投入，多做雪中送炭的工作，不让平均数掩盖部分群众的困难状况。市级财政预算安排民生支出210亿元，占财政支出的比重达到67.8%。重点办好11件市办实事。

　　坚持教育优先发展。加快推进寄宿制普通高中、幼儿师专等项目建设，新建、改扩建100所幼儿园，建设200所中小学标准化食堂，确保85%的普通中小学达到现代化学校标准。试行中小学教师聘用制，增聘教师3400名。加强与国内外高校的合作，支持驻青高校优势学科建设。承办好联合国教科文组织全球教育信息化大会。创新职业教育体制机制,推动职业教育产教融合、校企合作，扩大中职与高等院校一体化培养试点。支持民办教育发展。实施特殊教育提升计划。健全家庭经济困难学生资助体系，保障新市民子女平等接受教育。加强校车安全管理。

　　做好就业和社会保障工作。建立完善项目建设与培训就业联动机制,实行适龄就业人口全覆盖管理服务。实施技能青岛工程，完成技能帮扶、农村劳动力转移培训和职业农民技能证书培训4万人。实施大众创业工程，扶持创业1万人。新增就业25万人。促进同工同酬，保障外来务工人员合法权益。推进社会保险扩面征缴，加强社会保险与商业保险合作。稳妥推进机关事业单位养老保险制度改革。提高企业退休人员和城乡居民基本养老金标准。实施“三险合一”医疗保险新办法，居民社会医疗保险费政府补助标准提高到440元以上。城乡低保标准分别提高到每人每月620元和420元。完善大救助机制，发展慈善事业，让困难群众、因病致贫群众求助有门、受助及时。开工建设青岛市残疾人康复中心，提高残疾人托养服务补贴标准，托养困难家庭残疾人1万名。建筹1万套（户）保障性住房。实施1.5万户棚户区改造。

　　发展卫生计生事业。开工建设市人民医院、齐鲁医院二期、市立医院东院二期、青大附院东区综合病房楼和眼科医院。鼓励社会力量办医，积极引进国内外优质医疗资源。深化县级公立医院综合改革。启动公立医院法人治理结构和薪酬制度改革试点。推动医疗联合体建设，健全基层首诊、分级诊疗、双向转诊服务体系。完善全科医生执业方式和服务模式。扩大基本药物实施范围。建立统一的区域人口健康信息化平台。筹建公共卫生中心，基本公共卫生服务筹资标准提高到每人50元。加强妇幼医疗保健服务，提高优生优育水平。做好计划生育特殊困难家庭扶助工作。

　　完善养老服务体系。加快养老服务设施建设，鼓励社会力量举办医养结合型养老机构，新增养老床位6000张，千名老人床位数达到37张。推进社区老年人日间照料中心建设，日间照料服务实现城市社区全覆盖。支持发展老年护理、疾病康复和临终关怀事业，让广大老年人老有颐养、安享晚年。

　　支持驻青部队建设，做好国防教育和民兵预备役工作，争创全国双拥模范城“八连冠”。认真贯彻民族宗教政策，维护民族团结、宗教和睦。促进妇女事业全面进步和儿童健康成长。深化对台交流合作。完善大外事、大侨务工作机制。加快智慧档案馆建设。提升地方志编纂工作水平。增强仲裁公信力。促进人防与经济社会深度融合发展。提高气象服务和灾害防御水平。

　　（八）推动法治社会建设，维护社会和谐稳定。大力弘扬法治精神，深化社会专项治理，推进社会治理体系和治理能力现代化，率先建设法治城市。

　　增强全民法治观念。深入开展法制宣传“双五”建设和“法律六进”活动，引导群众自觉守法、遇事找法、解决问题靠法。完善公务人员学法用法制度。加强青少年普法工作。建立行政执法人员以案释法制度。加强社会诚信体系建设，初步建成法人和自然人公共信用信息数据库，健全公民和组织守法信用记录，依法公开相关公共信用信息，完善守法诚信褒奖机制和违法失信行为惩戒机制。

　　提高社会治理法治化水平。坚持系统治理，促进政府治理和社会自我调节、居民自治良性互动。逐步剥离社区和居委会经济职能。改革社会组织管理体制，完善政府购买服务机制，发挥社会组织在社会治理中的积极作用。坚持依法治理，完善公共法律服务体系，扩大法律援助范围。推进访诉分离，引导群众依法逐级反映诉求，努力及时就地解决信访问题。坚持综合治理，搭建基层综合服务管理平台，推行城乡综合治理网格化，提高社区组织化程度，建成社区便民服务站3000处。坚持源头治理，畅通利益诉求表达渠道，健全社会矛盾纠纷预防化解机制，引导群众理性表达诉求，依法维护权益。

　　深化重点领域专项治理。贯彻落实新修订的《安全生产法》，加强安全设施建设，健全事故隐患排查整治和处罚机制，强化责任落实，促进安全发展。严格实施农业投入品监管和产地准出制度，开展食品安全定性定量检测6.5万批次以上，保持对食品药品安全违法犯罪行为的高压态势，争创国家食品安全城市。优化交通组织，发展智能交通，增设公交专用道40公里，创建公交都市。加快停车场建设，增划限时停车泊位5000个，规范停车和收费管理。推行数字化城管，推进环卫作业市场化，深入整治占路经营、露天烧烤、违规养犬、违章建筑等热点难点问题。加强平安青岛建设，完善立体化社会治安防控体系，抓好“天网”工程建设和应用，加强社区警务、治安志愿者队伍建设，依法严厉打击违法犯罪活动。落实禁毒责任，最大限度减少毒品的社会危害。做好社区矫正工作。加强应急平台一体化建设，开展应急安全文化年活动，强化应急演练，增强全社会防灾救灾能力。

　　三、加快推进法治政府建设

　　坚持在法治轨道上开展工作，切实提高政府工作人员的法治意识和依法行政能力，建设职能科学、权责法定、执法严明、公开公正、廉洁高效、守法诚信的法治政府。

　　（一）坚持科学民主依法决策。坚决执行人大及其常委会的决议、决定，坚持重大事项向人大报告、向政协通报制度，自觉接受人大法律监督、工作监督和政协民主监督。切实做好人大代表建议、意见及政协提案办理工作。完善协商民主制度，认真听取各民主党派、工商联、无党派人士和人民团体的意见建议。坚持市政府领导联系服务代表、委员制度。健全依法决策机制，严格规范行政决策程序，完善重大决策合法性审查机制和社会稳定风险评估机制。健全政府法律顾问制度。加强智库建设，完善决策咨询制度。继续开展“三民”活动，发挥网络在线问政、行风在线、政务服务热线等互动平台作用，及时回应群众关切，主动接受舆论监督和公众监督。

　　（二）深入推进依法行政。全面推行行政权力清单和责任清单制度,确保法定职责必须为、法无授权不可为。加大政务公开力度，全面公开政府职能、法律依据、实施主体、职责权限、管理流程、监督方式等事项。做好地方性法规草案提请审议和政府规章制定工作。严格规范公正文明执法，探索建立行政执法全过程记录制度，健全行政裁量权基准制度，落实行政执法责任制。加大关系群众切身利益的食品药品安全、公共卫生、安全生产、环境监管、民生保障等重点领域执法力度，依法惩处各类违法行为。加强行政执法队伍建设。各级政府工作人员都要牢记法律红线不可逾越、法律底线不可触碰，带头遵守法律，带头依法办事。

　　（三）不断提高服务效能。坚持联系群众、联系企业、联系村居等各项制度，提高服务群众、服务企业、服务发展的能力和水平。发扬“钉钉子”精神和踏石留印、抓铁有痕的作风，进现场、解难题，确保重大决策、重点工作、民生实事不折不扣落到实处。加大庸懒散慢拖瞒治理力度,坚决纠正不作为、乱作为，坚决克服懒政、怠政，坚决惩处失职、渎职。发挥电子政务平台作用，建设全市一体化政务信息系统，完善网上便民服务体系，推进公共信息资源向社会开放。加快审批信息资源共享库建设。健全政府目标绩效考核指标体系和考评机制，构建常态化督查机制，建立第三方评估制度，推动各项工作加速提升创新增效落实。

　　（四）切实加强廉政建设。认真落实十八届中央纪委五次全会和国务院第三次廉政工作会议精神，把守纪律讲规矩摆在更加重要的位置，深入践行“三严三实”，驰而不息纠正“四风”，始终做到心中有党、心中有民、心中有责、心中有戒。各级政府和政府各部门党组（党委）要严格落实党风廉政建设主体责任，一级抓一级，做到守土有责、守土负责、守土尽责。各级领导干部要认真履行“一岗双责”，对职责范围内的党风廉政建设切实负起领导责任。制定出台市政府落实廉政责任、加强政府系统廉政建设的意见。坚决执行厉行节约规定，严格控制一般性支出，确保财政供养人员和“三公”经费只减不增。完成公务用车制度改革。推进惩治和预防腐败体系建设，强化对行政权力的制约和监督。围绕权力集中的部门和岗位，实行分事行权、分岗设权、分级授权，定期轮岗，强化内部流程控制，防止权力滥用。完善审计制度，对公共资金、国有资产、国有资源和领导干部履行经济责任情况实行审计全覆盖。加强对政府部门依法履职情况的监督，强化行政问责。坚持零容忍态度惩治腐败，坚决查处违法违纪案件，推动廉洁青岛建设，促进干部清正、政府清廉、政治清明。

　　各位代表，面对新常态，实现新发展，任务光荣而艰巨。让我们紧密团结在以习近平同志为总书记的党中央周围，在中共青岛市委的坚强领导下，凝心聚力，开拓进取，努力做好全年各项工作，为加快建设宜居幸福的现代化国际城市作出更大贡献！
